# Deciphering the impact of intra-tumoral bacterial infiltration on multi-omics profiles in low-grade gliomas

**DOI:** 10.3389/fonc.2025.1582068

**Published:** 2025-06-18

**Authors:** Wenshu Li, Zixiang Zhu, Longyuan Li, Xin Wu, Jiaxuan Li, Yi Zhou, Lingwen Gu, Pranathi Vittal, Zhouqing Chen, Zhong Wang, Lingchuan Guo

**Affiliations:** ^1^ Department of Pathology, The First Affiliated Hospital of Soochow University, Soochow University, Suzhou, Jiangsu, China; ^2^ Department of Neurosurgery & Brain and Nerve Research Laboratory, The First Affiliated Hospital of Soochow University, Soochow University, Suzhou, China; ^3^ Soochow University Medical College, Soochow University, Suzhou, China

**Keywords:** intra-tumoral bacterial, glioma, LGG, machine learning, immune checkpoint

## Abstract

**Background:**

Low-grade gliomas (LGGs) exhibit diverse bacterial infiltrations. This study delves into the intricate relationship between microbial infiltration in glioma samples and tumor multi-omics characteristics, aiming to elucidate its impact on tumor behavior and patient prognosis.

**Methods:**

We included low-grade glioma (LGG) samples from The Cancer Genome Atlas (TCGA) as analysis cohort and used LGG tumor samples from patients who underwent surgical treatment as validation cohort. For the TCGA samples, utilizing advanced machine learning algorithms, this study identified distinct patterns of bacterial infiltration within the LGG population and constructed a prognostically relevant intra-tumoral bacteria risk model (PRIBR Index). For the clinically derived samples, we performed 16S rRNA sequencing, bulk RNA sequencing, and proteomics analysis. Subsequently, the samples were stratified into high-risk and low-risk groups. We then explored clinical information, tumor microenvironment, methylation status, and sensitivity to targeted therapies between these groups to elucidate the impact of varying bacterial infiltration levels on glioma behavior.

**Results:**

A total of 32 common differentially expressed genes were identified between the TCGA-LGG samples and the clinical samples when comparing the high-risk and low-risk groups. The high-risk group demonstrated elevated bacterial infiltration levels, which were associated with increased infiltration of inflammatory factors. Patients in this group exhibited shorter survival periods, potentially attributable to the heightened expression of negative immune checkpoints. Predictive analysis for targeted drugs indicated that certain agents might achieve a lower half maximal inhibitory concentration (IC50) in the low-risk group compared to the high-risk group. Furthermore, while no significant differences were observed in tumor mutation burden or copy number variation between the two groups, the high-risk group showed increased methylation levels across multiple pathways.

**Conclusion:**

These findings offer new insights into the biological characteristics of gliomas and provide novel avenues for exploring new therapeutic approaches, bringing fresh perspectives to the field of intra-tumoral bacteria.

## 1 Introduction

Gliomas are the most common primary malignant tumors of the central nervous system (CNS), which account for about 30% of all CNS tumors and about 80% of malignant tumors (1, 2). Based on clinical and molecular pathology, the 2021 World Health Organization (WHO) Classification of Tumors of the Central Nervous System (5th edition) has classified adult-type diffuse gliomas into three types, including astrocytoma, IDH-mutant; oligodendroglioma, IDH-mutant and 1p/19q-deleted; and glioblastoma, IDH-wildtype (3). Low-grade gliomas (LGGs) compose 6% of primary tumors of the CNS in adulthood (4), with median survival times ranging from 5.6 to 13.3 years based on tumor histopathologic features (5–7). Glioblastoma (GBM) is the most prevalent high-grade glioma (HGG), which is among the deadliest malignant solid tumors, exhibiting a median survival time of less than 2 years (8). Thus, the current status of glioma treatment and prognosis is not deemed satisfactory. On one hand, although surgery is currently recognized as the primary treatment option, glioma cells are highly invasive and proliferative, making it difficult to remove tumor cells completely by surgery (9, 10); on the other hand, the blood-brain barrier (BBB) limits the efficacy of chemotherapeutics and monoclonal antibody agents, including lomustine, carmustine, and bevacizumab (11). Over the last few years, immune response-based immunotherapies, including adoptive cell transfer (ACT) and immune checkpoint blockade (ICB), have revolutionized the effectiveness of treatments for patients with tumors (12). In addition, the discovery of potential targets, including Alkylating agent, Tyrosine kinase receptor pathway, BRAF mutation, NF1, and IDH-mutation, also has offered more viable options for targeted therapy of gliomas (13, 14). However, to date, no novel therapeutic agent has received regulatory approval for glioma, and immune checkpoint inhibitors—which have shown remarkable efficacy in various cancers—have likewise failed to demonstrate significant benefits in clinical trials for glioma (15, 16). As such, glioma investigation and treatment have emerged as a popular clinical challenge.

There is increasing evidence that the microbiome in the human body is strongly associated with various tumors, including hepatocellular carcinoma (17), pancreatic ductal adenocarcinoma (18), breast cancer (19), and lung cancer (20). Numerous studies have shown that the gut microbiome may play an indispensable role in the pathogenesis and pathophysiology of tumors (21, 22). Recent studies have shown intratumor microbes are also significantly associated with tumorigenesis and prognosis. A large cohort study of intratumor microbes in 2020 provided a comprehensive characterization of the intratumor microbiome (23). Seven common tumors were included in the study, and it is worth mentioning that GBM was included. The presence of microbial DNA in human tumors was confirmed by microbial 16S rDNA real-time quantitative polymerase chain reaction (qPCR), and then antibodies against microbial lipopolysaccharide (LPS) and microbial 16S rRNA were exhibited that the microbiome was mainly localized in cancer cells and immune cells. Besides, immunofluorescence (IF) staining demonstrated that microbiome was found in CD45+ immune cells, which suggested that they might have an impact on the immune state of the tumor microenvironment (23). In terms of the mechanisms triggering tumorigenesis, intratumor microbes can directly cause DNA damage besides metabolite pathways, leading to tumor formation and progression (24). Furthermore, relevant studies have shown that intratumor microbes metabolize chemotherapy drugs and lead to chemotherapy resistance (25). Thus, intratumor microbes also have a significant impact on cancer development and progression.

Nevertheless, limited research has emerged on intratumor microbes and gliomas, which may be related to the common belief that the brain is sterile. Recent studies on Alzheimer’s disease had shown that microbes are also integral to the brain tissue under non-inflammatory and non-traumatic conditions (26, 27). A 2023 immunology study by Roland Martin’s team found that HLA molecules in glioblastoma present bacterial-specific peptides. This peptide of microbial origin is recognized by tumor-infiltrating lymphocytes (TILs), triggering a respond to tumor-derived target peptides (28). However, the potential relationship linking microbes within gliomas to tumor development is unclear. To investigate the association between the two and the impact on the overall survival of patients, we conduct this article to provide a theoretical basis for the diagnosis and treatment of gliomas.

## 2 Methods

### 2.1 Data collection and standardization

The mRNA counts and TPM format transcriptome of LGG samples are sourced from the TCGA database (TCGA-LGG, https://portal.gdc.cancer.gov/), along with downloaded genomics and clinical information for analysis. Methylation β-values of different pathway genes in different samples are obtained from cBioPortal (www.cbioportal.org). The microbiome abundance matrix of TCGA-LGG samples is sourced from Bacteria in Cancer (BIC, http://bic.jhlab.tw/) (29), which utilizes TCGA miRNA sequencing data to calculate the abundance of bacteria within tumors by comparing reads not mapped to the human genome against a bacterial reference. All data, except for necessary removal of NA values or log transformation, have not undergone any other modifications.

### 2.2 Collection of clinically sourced LGG samples and 16S rRNA sequencing

The LGG samples included in this study were obtained from patients who underwent surgical resection at the Department of Neurosurgery, The First Affiliated Hospital of Soochow University, between May 2023 and November 2023. Both patients and their families were informed preoperatively that part of the tumor samples might be used for scientific research, and written informed consent was obtained. After tumor resection, the samples were collected in sterile cryogenic vials (Bioteke, China) under sterile conditions. The entire collection process was conducted on a sterile workbench, after which the samples were transferred to a -80°C freezer. The next day, the samples were moved to long-term storage in liquid nitrogen. Once the pathological diagnosis confirmed the tumors as LGG, the samples were included in the study. For this research, the samples were sent to GENEWIZ (Suzhou, China) for 16S rRNA sequencing. The V3 and V4 hypervariable regions of the prokaryotic 16S rRNA gene were amplified using PCR primers designed by GENEWIZ (Suzhou, China). The forward primer 338F (including the sequence “ACTCCTACGGGAGGCAGCAG”) and the reverse primer 806R (including the sequence “GGACTACHVGGGTWTCTAAT”—with portions of the sequence withheld for confidentiality) were employed for the amplification. Subsequently, index adapters were attached to the PCR products via an additional PCR step to enable next-generation sequencing (NGS). The resulting PCR product library was validated on a 1.5% agarose gel, which confirmed a target fragment of approximately 600 bp. For bioinformatics processing, raw sequencing data were optimized using Cutadapt (v1.9.1), Vsearch (v1.9.6), and Qiime (v1.9.1) through the following steps: 1: Paired-end reads were aligned and merged based on overlapping regions of at least 20 bp, with sequences containing ambiguous bases (“N”) removed; 2: Adapter sequences were trimmed, bases with quality scores below 20 at both ends were removed, and sequences shorter than 200 bp were discarded; 3: The merged and filtered sequences were compared against a database to identify and remove chimeric sequences, yielding the final high-quality dataset.

### 2.3 LGG microbial characteristics and establishment of machine learning prognostic models

Using R software (version 4.3.2), we first included survival information and microbial abundance of all LGG samples for single-factor Cox regression analysis (survival package, version 3.5-7). Bacteria with p < 0.05 were selected for subsequent multivariate Cox regression analysis (survival package, version 3.5-7), using p < 0.05 as the threshold. Subsequently, a combination of 60 machine learning algorithms was employed to establish survival-related models for the abundance of screened bacteria and sample survival information in each sample, ranked by the area under the ROC curve. The optimal combination was selected to construct the LGG microbiota prognosis prediction model. Validation was conducted using Kaplan-Meier (KM) curves, time-dependent ROC curves, calibration curves, and decision curves analysis. Finally, based on the abundance of bacteria included in the model in each sample, a risk score was calculated. Using the median score as the threshold, LGG samples were divided into PRIBR Index.

### 2.4 Proteomic sequencing

For this research, the samples were sent to GENEWIZ (Suzhou, China) for proteomic sequencing. Frozen tissue samples are cryogenically ground and lysed to extract total proteins, which are quantified by BCA. Proteins are reduced, alkylated, precipitated, and then digested with trypsin. The resulting peptides are desalted using SPE, separated via nano-LC, and analyzed by DIA on a Bruker timsTOF Pro mass spectrometer. Finally, protein identification and quantification are performed using Spectronaut Pulsar software.

### 2.5 Construct nomogram by incorporating clinical characteristics

We collected clinical information from LGG samples and constructed a nomogram model incorporating risk score, age, gender, 1p/19q chromosomal co-deletion status, WHO grading, and MGMT methylation status. This model aims to examine whether the risk score can serve as an independent predictor for prognosis, predicting the survival rates of glioma patients at 1, 3, and 5 years. Validation was performed using time-dependent ROC curves and decision curve analysis.

### 2.6 Analysis of mRNA expression difference between high and low risk groups

We utilized the “DESeq2” package (version 3.58.1) to conduct differential analysis of mRNA expression levels between the high and low-risk groups. We applied adjusted p-values < 0.05 and absolute log fold change (LogFC) > 1 as filtering criteria. Visualization was performed using the ggplot2 package (version 3.4.4).

### 2.7 Enrichment analysis

We employed the R, utilizing the org.Hs.eg.db package (version 3.1.0) for gene annotation from the GO database, and the KEGG REST API (https://www.kegg.jp/kegg/rest/keggapi.html) for gene annotation from the KEGG database. Enrichment analysis was conducted using the clusterProfiler package (version 3.14.3), with a minimum gene set of 5 and a maximum gene set of 5000. Visualization was performed for the top ten pathways with a P value < 0.05.

### 2.8 Analysis of tumor microenvironment

We applied the ESTIMATE method and the EPIC method to calculate the tumor microenvironment components of LGG samples, quantifying the infiltration abundance of each type of cell. ESTIMATE is an algorithm that leverages mRNA sequencing data to predict cell types, enabling the assessment of tumor cell purity, stromal fraction, and overall immune cell infiltration in samples (30). In contrast, EPIC estimates the composition of cell types using a reference gene expression matrix, focusing on the proportions of various immune cell types within the sample and evaluating their absolute scores (31). Subsequently, we utilized the R “pheatmap” package (version 1.0.12) to visualize the tumor microenvironment components between the two groups.

### 2.9 Tumor mutation burden and copy number variation

We organized the Masked Somatic Mutation files and Copy Number Variation (CNV) files obtained from the TCGA database for LGG samples. We employed the R software package maftools (version 2.18.0) to visualize the tumor mutation burden (TMB). Furthermore, we utilized the GISTIC2.0 functionality on the GenePattern website (https://cloud.genepattern.org/) for Copy Number Variation analysis. Subsequently, we used the R software package maftools (version 2.18.0) to visualize TMB and CNV.

### 2.10 Drug sensitivity analysis

We downloaded the tumor drug sensitivity v2 dataset (GDSC2, pSet name: GDSC_2020 (v28.2)) using the “pharmacoGx” package. This package allows for efficient annotation of cell lines, drug compounds, and molecular features, facilitating comparison and integration of different drug genomics datasets. The “OncoPredict” package is utilized for predicting drug responses in cancer patients. The calcPhenotype function predicts the half-maximal inhibitory concentration (IC50) of drugs in glioma patients by fitting a ridge model, with the training set consisting of tissue gene expression profiles and cancer cell line IC50 values for GDSC2 and LGG drugs, and the test set comprising RNA-seq profiles of TCGA glioma patients. We employed the Wilcoxon signed-rank test to analyze differences in predicted IC50 between low- and high-risk groups, using p < 0.05 as the significance threshold.

## 3 Results

### 3.1 A variety of bacteria can be identified in low-grade gliomas, and the abundance of multiple bacteria is correlated with patient survival

**Figure 1:**
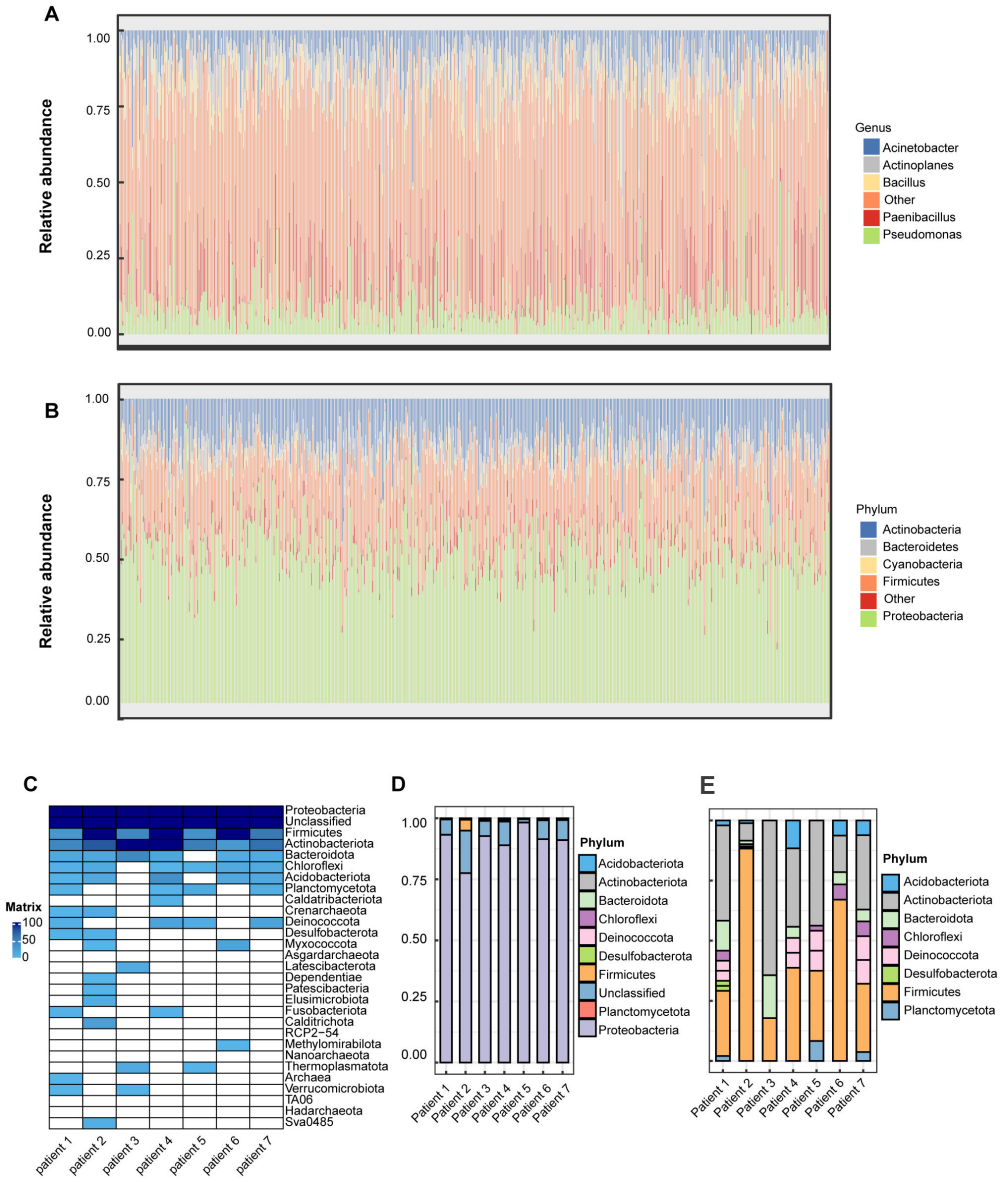
Relative abundance of different microbial communities in TCGA-LGG samples using stacked bar charts. Relative abundance was obtained by summing the readings of species that passed all the screens and belonged to the same phylum/genus. **(A)** Bacterial phylum-level composition of TCGA-LGG patient samples. The top five genus names are listed in the legend: Pseudomonas, Paenibacillus, Acinetobacter, Actinoplanes, and Bacillus. **(B)** Genus-level composition of bacteria in the Proteobacteria phylum in TCGA-LGG patient samples. The top five genus names are listed in the legend: Proteobacteria, Firmicutes, Actinobacteria, Bacteroidetes, and Cyanobacteria. **(C)** Heatmap showing the infiltration of the top 30 most abundant bacterial phyla in clinically sourced LGG samples based on 16S rRNA sequencing, with gradient colors indicating the sequence counts of each taxon after z-score normalization. **(D)** Proportional infiltration of the top ten most abundant bacterial phyla in clinically sourced LGG samples based on 16S rRNA sequencing. **(E)** Proportional infiltration of the top ten most abundant bacterial phyla in clinically sourced LGG samples based on 16S rRNA sequencing, after excluding Proteobacteria and unclassified bacterial phyla.

The results obtained from the BIC database revealed that in TCGA-LGG samples, the most abundant bacterial phylum is Proteobacteria (**Figure 1A**). Within the *Proteobacteria* phylum, *Pseudomonas*, belonging to the *Pseudomonadaceae* family, ranked first in abundance across the samples and was detectable in most tumor samples (**Figure 1B**). After conducting 16S rRNA sequencing on the clinically sourced LGG samples, we found that the microbial phyla *Proteobacteria, Firmicutes, Actinobacteria, and Bacteroidetes*—previously identified as the top five in abundance in TCGA—were also detectable in our clinical samples, we visualized the sequence counts of the top 30 most abundant bacterial phyla (**Figure 1C**). Similar to the TCGA-derived samples, *Proteobacteria* were present in our clinical samples in high proportions (**Figure 1D**). However, one notable difference is that the proportion of *Proteobacteria* in our samples was exceedingly high. Given that *Proteobacteria* are a common bacterial phylum, we had to consider the possibility of sample contamination. After excluding *Proteobacteria*, the most abundant identified bacterial phylum in our samples was *Firmicutes* (**Figure 1E**).

It’s puzzling that multiple bacterial infiltrations were detected in nearly all samples. Typically, the intracranial environment is considered absolutely sterile, a fundamental principle upheld during neurosurgical procedures (32). Despite rigorous environmental disinfection, opening the dura mater during surgery inevitably exposes brain tissue to airborne bacteria. Neurosurgeons commonly employ prophylactic antibiotics to prevent intracranial infections, yet the risk remains significantly high (33). Therefore, the presence of bacteria in primary intracranial tumors may confound most neurosurgeons. Indeed, previous studies have demonstrated that bacteria can penetrate the blood-brain barrier through mechanisms involving cellular invasion or infecting phagocytic cells (34, 35). Building upon this foundation, numerous studies have explored targeted drug delivery using Trojan horse mechanisms that exploit bacterial traversal of the blood-brain barrier (36, 37). Based on this theoretical framework, we endeavor to analyze whether microbial presence within tumors may offer potential benefits in the treatment of LGG. Survival-associated single-factor COX analysis identified the abundance of 55 bacterial genera out of 1618 genera correlated with patient survival (**Supplementary Table S1**). Subsequent multivariate Cox analysis further narrowed down the list to 33 bacterial genera, of which 8 belonged to the *Proteobacteria* phylum (*Oceanimonas, Shewanella, Burkholderia, Rahnella, Brucella, Edwardsiella, Pelagibacterium, Acidovorax*) (**Supplementary Table S2**).

### 3.2 The LGG microbial-related prognosis model was constructed by selecting the optimal machine learning model

**Figure 2:**
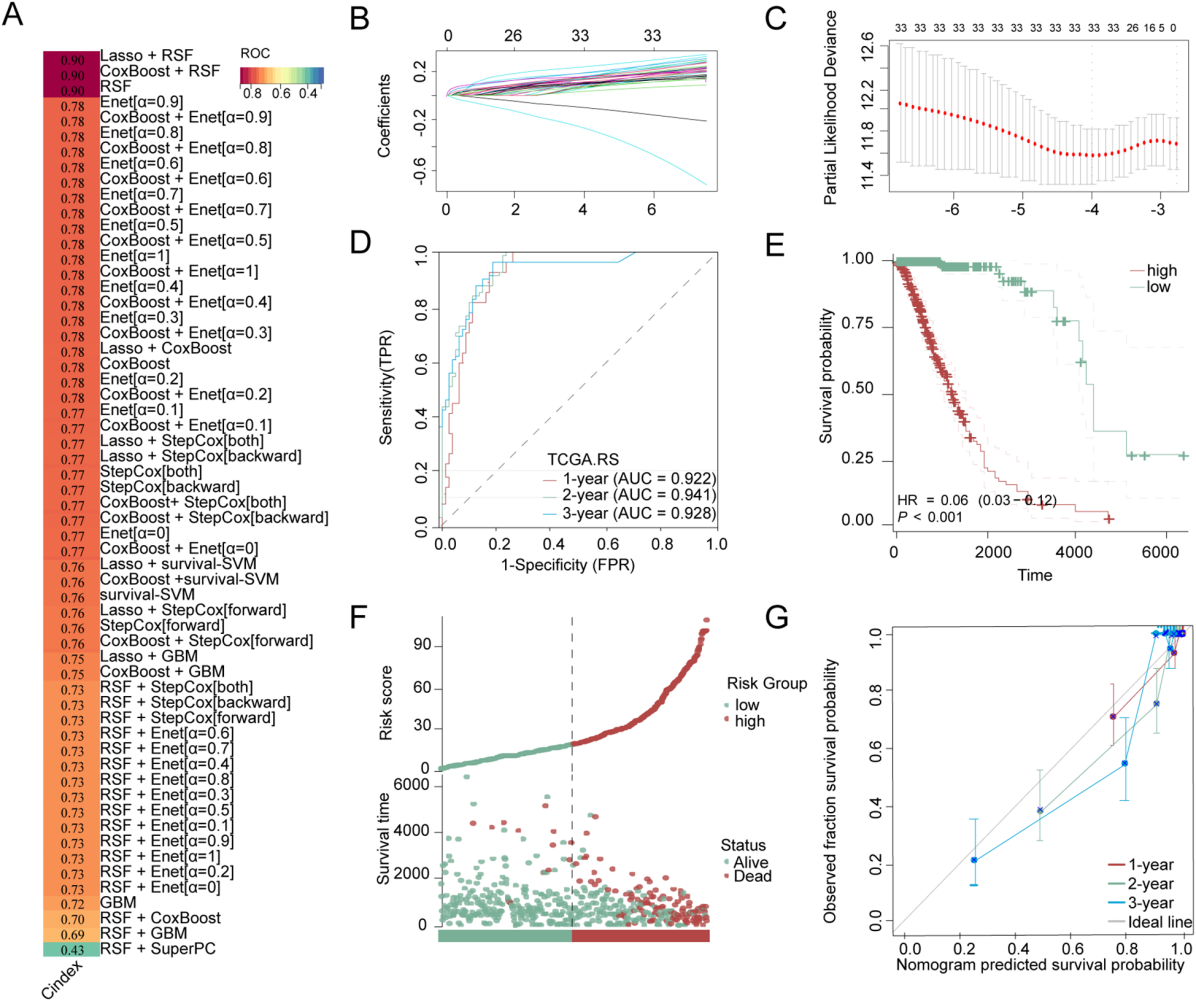
Construction of LGG microbial-related prognosis models and evaluation of model accuracy. **(A)** Stacked bar charts obtained by sorting the areas under the ROC curves of LGG microbiota-related prognostic models constructed by combinations of 60 different machine learning algorithms, with the dark red bars corresponding to the three algorithms employed with the highest ROC curve areas: the RSF+Lasso algorithm, the CoxBoost+RSF algorithm, and the RSF algorithm, with gradient colors representing the C-index values. **(B)** The RSF+Lasso algorithm coefficient profiles of 33 bacterial genera in TCGA-LGG dataset **(C)** The log (lambda) sequence plot of the 33 bacterial genera using RSF+Lasso algorithm regression. **(D)** The time-dependent ROC analysis of the RSF+Lasso algorithm risk score in TCGA-LGG. **(E)** Kaplan-Meier for groups with high-risk or low-risk scores. **(F)** Clinical outcome distribution and PRIBR Index distribution of LGG patients. **(G)** The calibration plot for predicting the 1-year, 2-year, and 3-year survival time.

**Figure 3:**
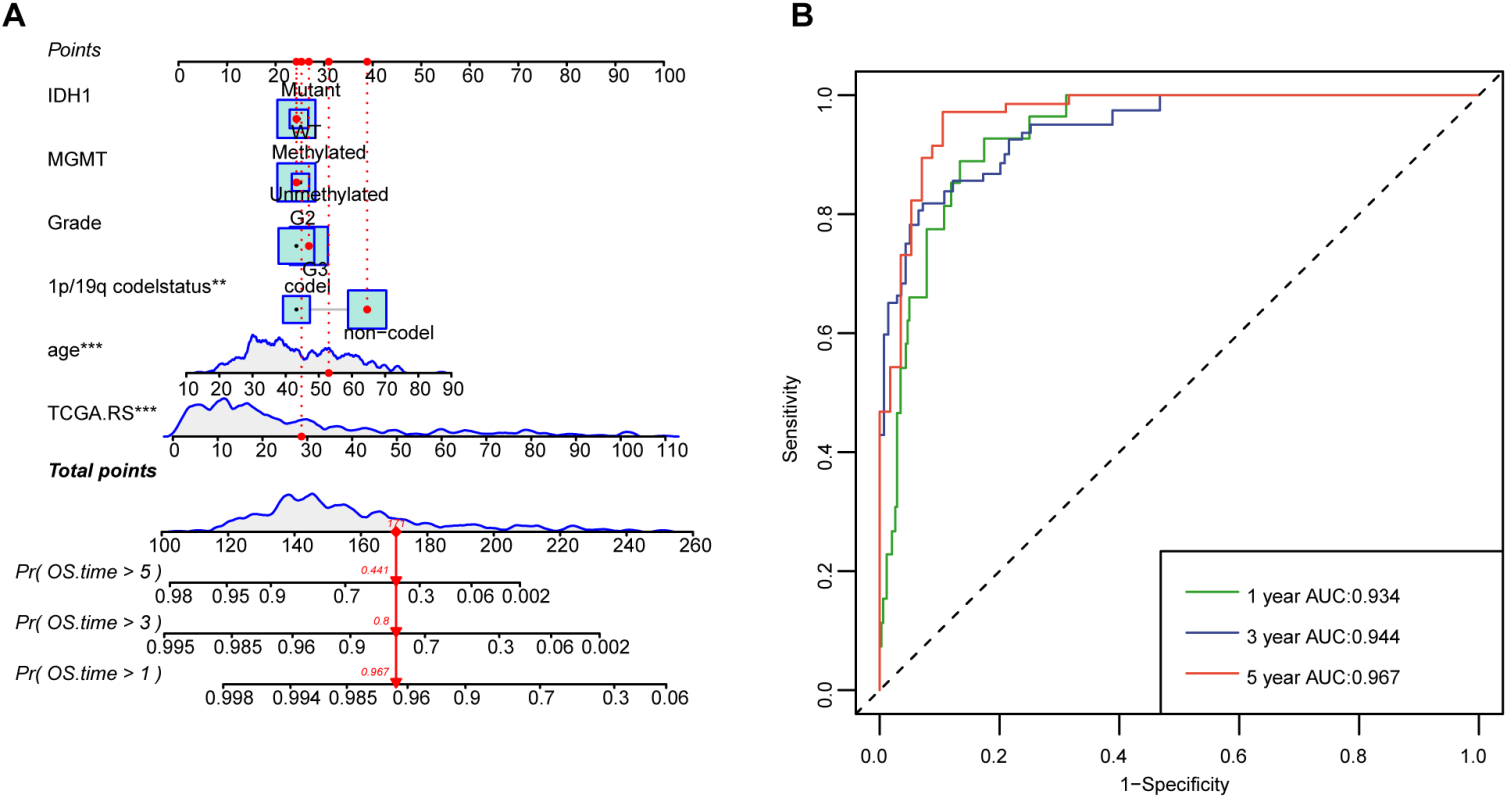
Nomograms with the risk score are displayed to predict the survival time of LGG patients. **(A)** The nomogram based on risk scores was constructed to predict the 1-year, 3-year, and 5-year survival time of glioma patients. **(B)** The corresponding ROCs illustrate the performance of the nomogram model with risk scores. **P < 0.01; ***P < 0.001.

We utilized 60 different combinations of machine learning algorithms and constructed an LGG microbiota-related prognostic model using the 33 bacterial genera selected from the previous multivariate COX analysis. By calculating the area under the ROC curve for each model and sorting them, the RSF+Lasso algorithm, CoxBoost+RSF algorithm, and RSF algorithm applied individually exhibited the highest ROC curve areas, approximately 0.90 (**Figure 2A**). Ultimately, we chose to construct the model using the top-ranked RSF+Lasso algorithm, which incorporated the abundance of all 33 bacterial genera (**Figures 2B, C**). Using time-dependent ROC analysis, we confirmed that our model remains accurate at the 1-, 2-, and 3-year time points (AUC > 0.80) (**Figure 2D**). Examination of the model revealed that the high-risk group had shorter survival time, a higher number of deaths (**Figure 2E**), and the PRIBR Index calculated based on this model demonstrated satisfactory accuracy in predicting the 1-year, 2-year, and 3-year survival of glioma patients (**Figures 2F, G**). After incorporating patient age, gender, 1p/19q chromosomal co-deletion status, MGMT methylation status, and WHO grading (**Supplementary Figure S1**), we constructed a nomogram to evaluate whether the risk score could serve as a reliable indicator for predicting prognosis. The results showed that the risk score had significant statistical significance in predicting patient survival time (p < 0.0001) (**Figure 3A**). Additionally, the nomogram exhibited ROC curves greater than 0.90 for predicting patient survival rates at 1 year, 3 years, and 5 years (**Figure 3B**).

### 3.3 A high abundance of bacteria is associated with cellular immune and inflammatory pathways in tumors

**Figure 4:**
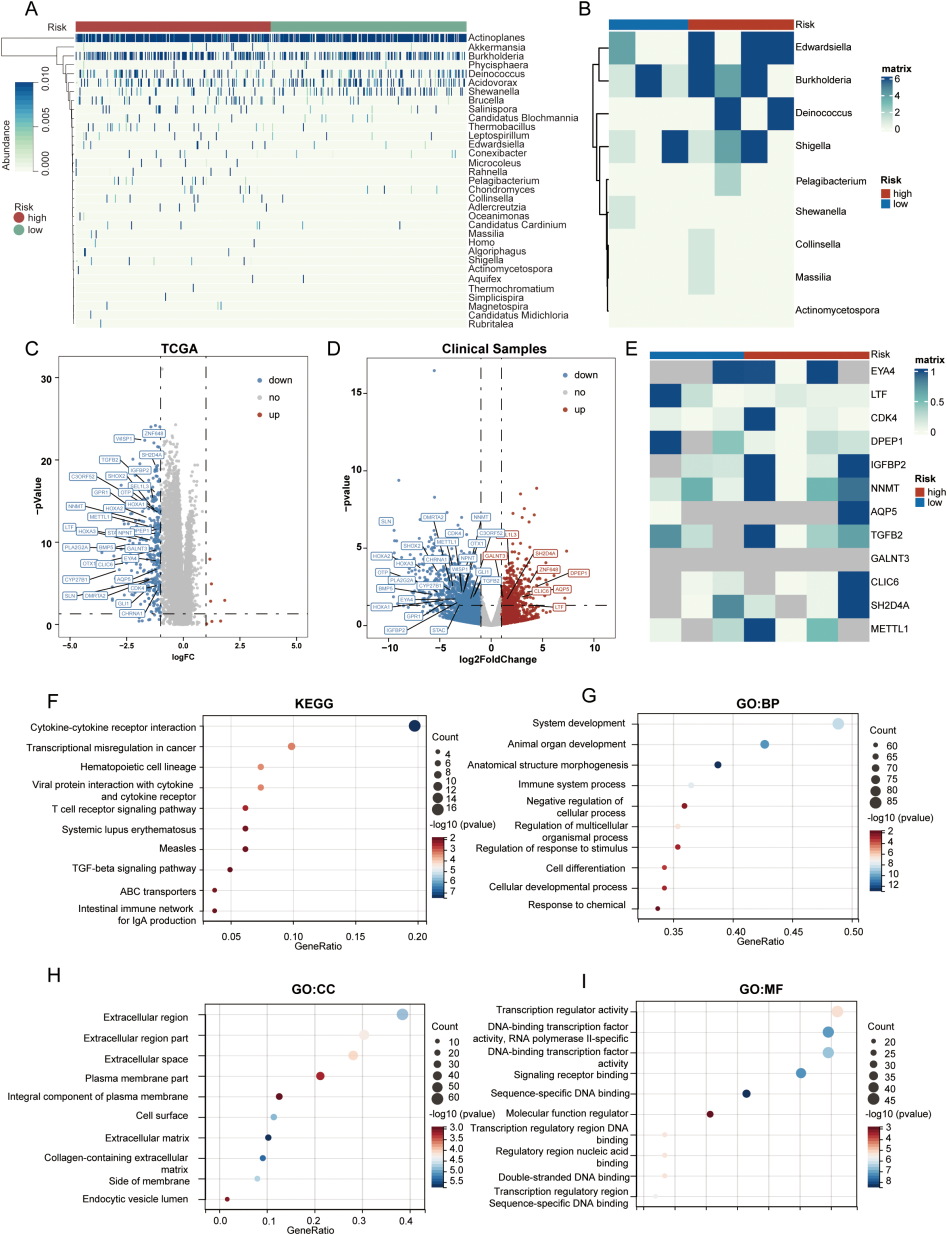
Cellular immune and inflammatory pathways in tumors of different PRIBR Index groups. **(A)** Heatmap showing the abundance distribution of 33 filtered bacterial genera in LGG samples from both high- and low-risk groups, with gradient colors indicating the infiltration abundance of each taxon after z-score normalization. **(B)** Heatmap displaying the infiltration abundance of 9 filtered bacterial genera measured in the clinical samples, with gradient colors indicating the infiltration abundance of each taxon after z-score normalization. **(C)** Volcano plot of differentially expressed genes between high- and low-risk groups in TCGA-LGG samples. **(D)** Volcano plot of differentially expressed genes between high- and low-risk groups in clinical samples, with mRNAs that are positively differentially expressed in both cohorts annotated [(logFC > 1, p < 0.05)]. **(E)** Heatmap illustrating the expression differences of the proteins translated from the 32 commonly expressed mRNAs in the proteomics analysis of clinical samples, where gray indicates that the protein was not detected, with gradient colors indicating the expression levels of each protein after z-score normalization. **(F–I)** Gene enrichment analysis of chemokines, T-cell signaling pathways, and other pathways related to cellular immunity and inflammation.

We presented a heatmap displaying the abundance distribution of the 33 bacterial genera included in LGG samples from the high-risk and low-risk groups. Within the high-risk subgroup of the TCGA cohort, several bacterial taxa—specifically *Brucella, Thermobacillus, Collinsella, Rahnella, and Chondromyces*—showed markedly greater inferred abundance. Correspondingly, the heat-map reveals a denser pattern of dark modules in these high-risk samples, signifying overall stronger microbial infiltration (**Figure 4A**). In the clinical sample cohort, our 16S rRNA sequencing detected 9 out of these 33 bacterial genera, and based on the abundance of these 9 genera, the samples were similarly classified into high-risk and low-risk groups (**Figure 4B**).

Subsequently, we conducted a differential analysis of mRNA expression levels between the two groups (**Supplementary Table S3**), selecting differentially expressed genes with an absolute log fold change (logFC) > 1 and an adjusted p-value < 0.05. Surprisingly, almost all of these genes exhibited higher expression trends in the high-risk group (**Figure 4C**). In addition, within the clinical sample cohort, we identified 32 mRNAs that were consistently differentially expressed across both the TCGA and clinical cohorts (**Figure 4D**).

In the proteomic sequencing of the clinical samples, although not all proteins corresponding to these 32 mRNAs were detected in every sample, the detected proteins still showed a similar expression trend. Notably, three genes that were highly expressed in the low-risk group are PCDHGB4, KRT14, and KRT6A. Among these, KRT14 and KRT6A belong to the cytokeratin family, a group of cytoskeletal proteins; downregulation of these proteins may indicate a reduction in the defensive capabilities of the barrier system (38).

Results from various enrichment analyses indicated that genes with higher expression in the high-risk group were enriched in pathways related to chemokines, T-cell signaling, and other cellular immunity and inflammation pathways (**Figures 4F–I**, **Supplementary Table S4**). Moreover, **Supplementary Figures S2B–E** displays the pathway enrichment of the 32 common differentially expressed genes identified in both cohorts, suggesting a correlation between high bacterial abundance and increased activity in these pathways within tumors. Considering that some genes exhibited opposite trends across the two cohorts, we conducted a subsequent enrichment analysis focusing solely on the genes with consistent trends. The results indicated that after excluding the genes with conflicting trends, there were no significant changes in the enriched pathways (**Supplementary Table S5**). Finally, after calculating the cellular components of the tumor microenvironment using the ESTIMATE and EPIC methods, we observed that macrophage infiltration—as well as the StromalScore, ImmuneScore, and ESTIMATEScore—were all significantly higher in the high-risk group (**Supplementary Figure S2A**).

### 3.4 The degree of immune cell infiltration in tumor microenvironment was different between PRIBR Index groups

**Figure 5:**
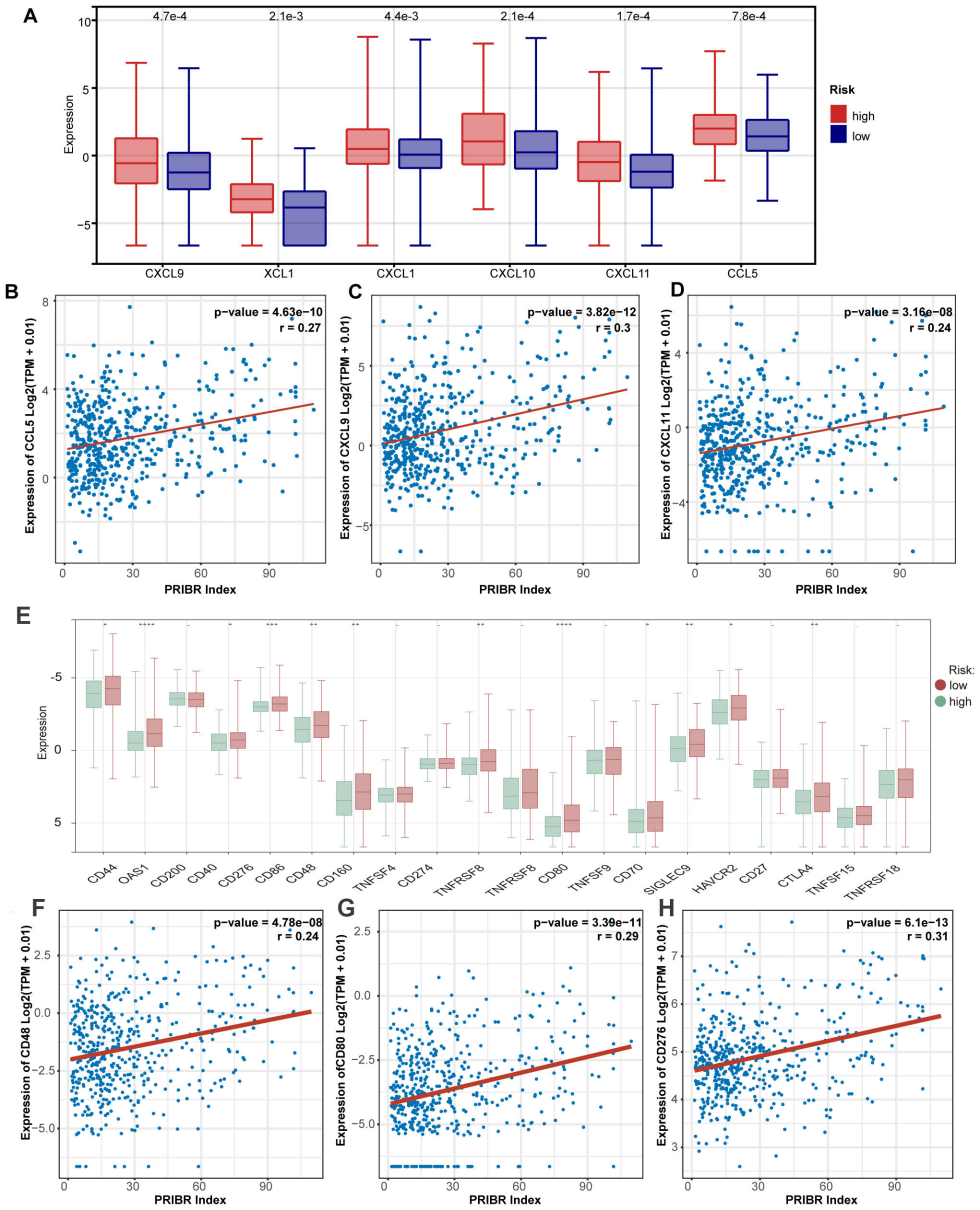
Different PRIBR Index groups showed different phenotypes. **(A)** Boxplot of the differential expression of chemokines between the high-risk and low-risk groups. **(B–D)** Correlation analysis between the risk score and expression of relative chemokines (CCL5, CXCL10, CXCL11). **(E)** Box plots are presented about the differential mRNA expression of various negative immune checkpoints between the high-risk and low-risk groups by the Kruskal–Wallis test. -, not significance, *P < 0.05; **P < 0.01; ***P < 0.001; ****P < 0.0001. **(F–H)** Correlation analysis between the risk score and expression of various negative immune checkpoints (CD80, CD276, CD48).

Subsequently, we used a heatmap to illustrate the differential expression of chemokines such as CCL5, CXCL10, and CXCL11 between the high-risk and low-risk groups (**Figure 5A**), showing higher expression patterns in the high-risk group. Moreover, there was a correlation between the risk score and these chemokines (**Figures 5B–D**), suggesting that bacteria present in tumors may jointly induce immune responses. Previous studies have indicated that microbiota in the gut or within tumors can promote antitumor immunity by inducing more severe inflammation or immune responses (18, 39). However, our earlier research showed that the abundance of these 33 bacteria genera associated with prognosis correlated with poorer outcomes in glioma patients. Considering the cold immune characteristics of gliomas (40), we attempted to analyze the differential mRNA expression of various negative immune checkpoints, including PDL1. The results showed that these negative immune checkpoints exhibited higher expression trends in the high-risk group (**Figure 5E**), and the expression levels of PDL1 (CD274), CD48, CD80, and CD276 were positively correlated with the risk score (**Figures 5F–I**). In our proteomic analysis, we demonstrated that the expression patterns of proteins translated from these mRNAs, as depicted in the heatmap, exhibited a similar trend (**Supplementary Figure S3A**). However, because these proteins were not measured across all samples, we incorporated glioblastoma mRNA and corresponding protein sequencing data from the CPTAC database. Correlation analysis revealed that the expression levels of CD48, CD276, and CD274 proteins in glioma were significantly correlated with their mRNA levels (r > 0.6, p < 0.05) (**Supplementary Figures S3B-D**). This may explain why the immune benefits brought about by bacteria in gliomas show a trend contrary to that seen in tumors in other locations.

### 3.5 The bacterial abundance in LGG was weakly associated with the mutant landscape of the tumor

**Figure 6:**
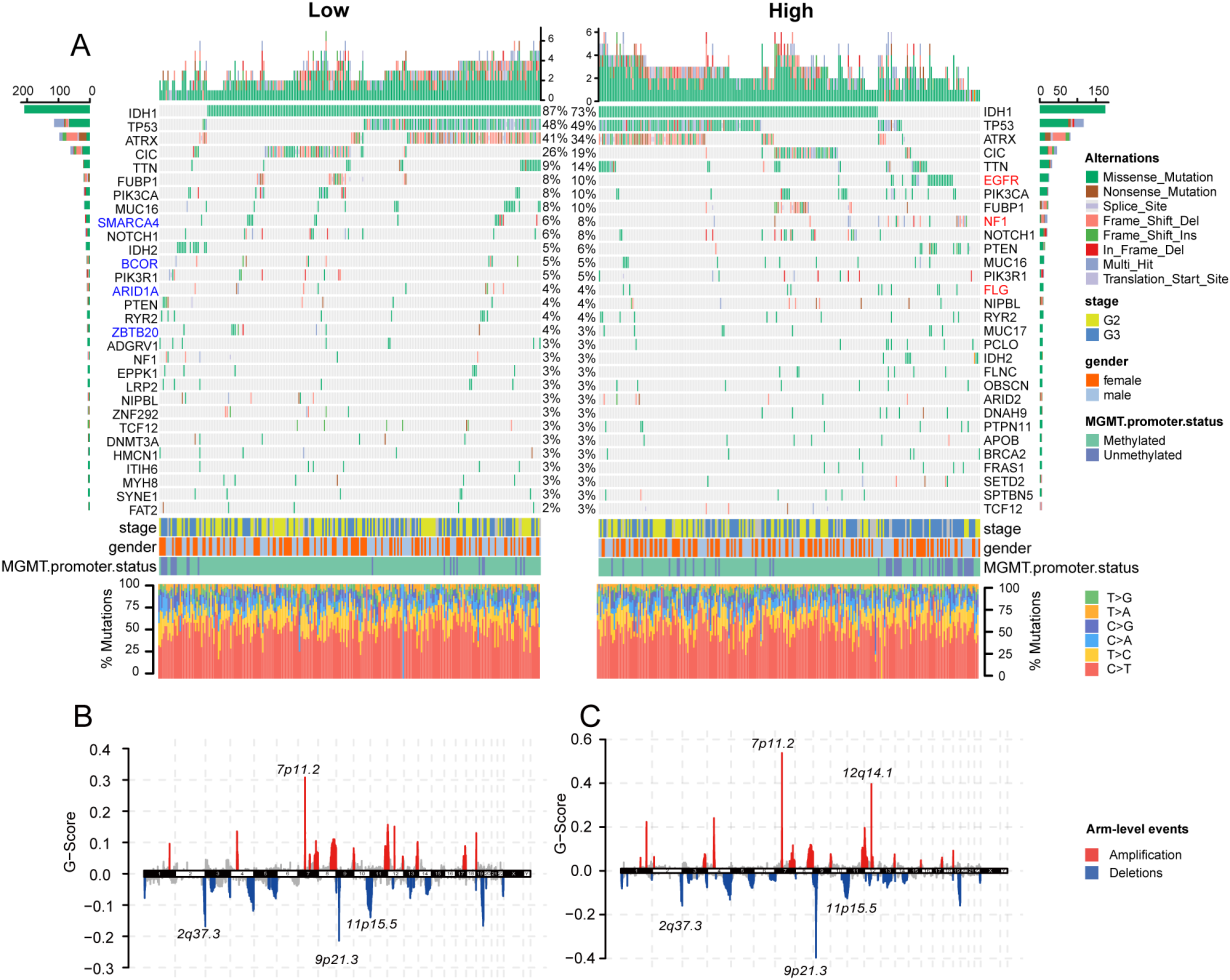
Association of bacterial abundance in LGG with tumor mutation. **(A)**. Waterfall plots exhibited top 30 genes mutation information in each sample in the high-risk and low-risk group. Column heatmap showed base-pair mutations in genes (genes with higher mutation frequencies in the low-risk group are highlighted in blue, while those more frequently mutated in the high-risk group are marked in red). **(B, C)** Chromosome plots demonstrated recurrent copy number variation (CNV) in the high-risk **(B)** and low-risk group **(C)**.

We attempted to analyze whether there were differences in gene mutations or chromosomal alterations between the high-risk and low-risk groups. However, the results showed that in the high-risk group, only the mutation probability of EGFR differed significantly (**Figure 6A**), while the mutation landscape of other genes was relatively similar. The mutation percentage is highest for IDH1, with 73% in the high-risk group and 87% in the low-risk group. This is because the samples included in TCGA are from a long time ago and did not utilize the 2021 WHO classification for central nervous system tumors to categorize glioma samples. As a result, some samples now considered to be glioblastomas were classified as grade 3 or grade 2 (3). Similarly, the results of CNV analysis did not reveal significant differences between the two groups (**Figures 6B, C**).

We selected genes involved in important pathways in the development of gliomas such as the WNT signaling pathway, p53 signaling pathway, TGFβ signaling pathway, and PI3K signaling pathway to examine their methylation levels. The results indicate that, in most cases, there were no significant changes in gene promoter methylation. However, there was a substantial increase in the percentage of complete methylation for several genes (**Supplementary Table S6**). This change ranged from approximately 10% to 15%. Previous studies have suggested that certain gut microbiota can influence epigenetic changes in cells (41). This result may explain why there are differences in mRNA expression levels among LGG samples with different bacterial abundances.

### 3.6 Different PRIBR Index groups exhibited varying drug sensitivities

**Figure 7:**
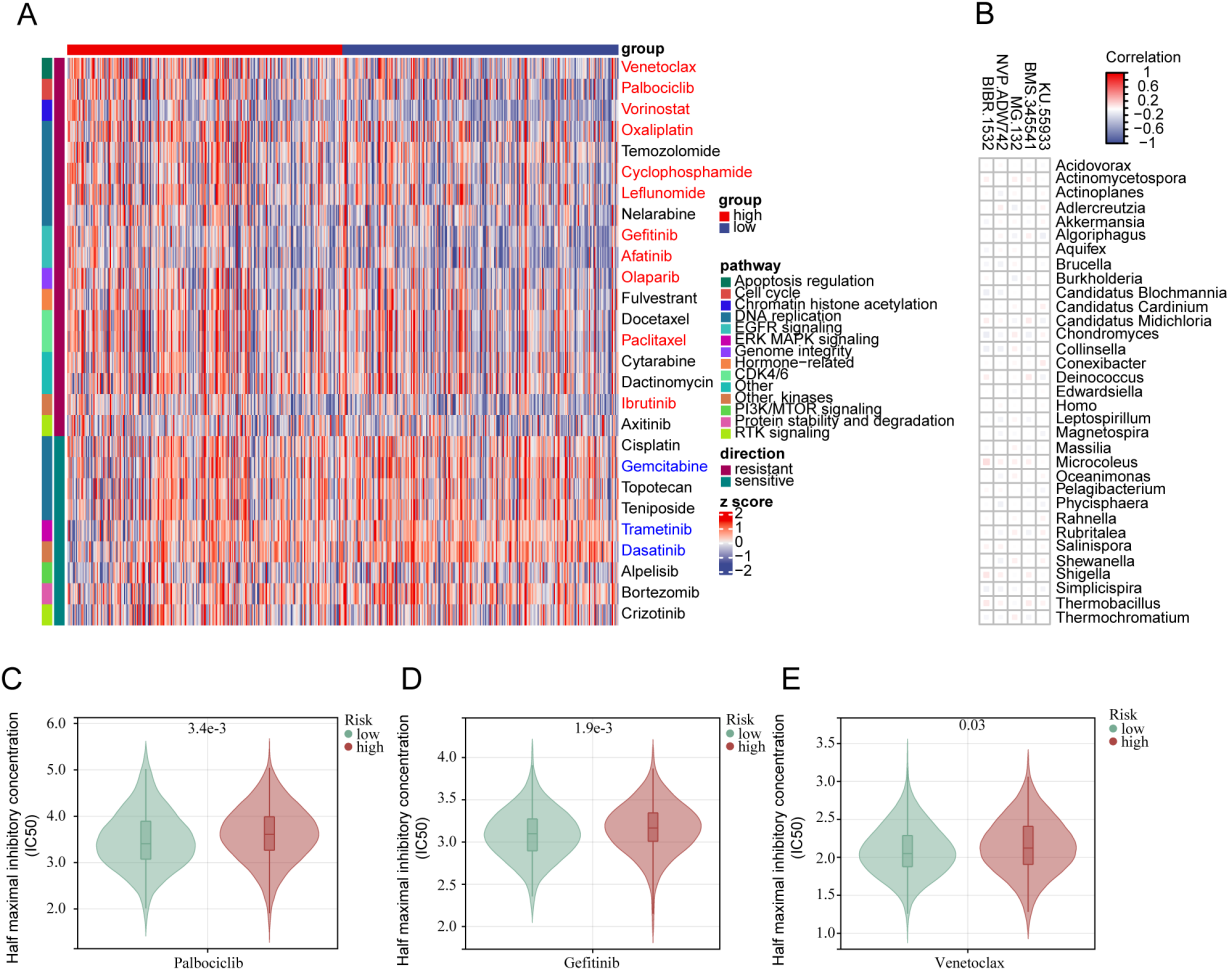
The PRIBR Index predicts patient sensitivity to targeted therapy: **(A)** Heatmap displaying 27 targeted therapeutic drugs exhibiting differences between the two groups, with annotations on the right indicating the targeted pathways for each drug (blue indicates drugs with an IC50 positively correlated with risk (correlation coefficient > 0.1 and p < 0.05), and red indicates drugs with an IC50 negatively correlated with risk (correlation coefficient < -0.1 and p < 0.05)). **(B)** Correlation analysis between 33 bacterial species and five specific targeted drugs. **(C–E)** Boxplots illustrating differences in IC50 values of different drugs between the two groups: Palbociclib **(C)**, Gefitinib **(D)**, Venetoclax **(E)**.

Using IC50 as the criterion for assessment, we attempted to predict drug sensitivity in all LGG samples. In the low-risk group, several targeted drugs were identified to have lower IC50 values (**Figure 7A**), including Gefitinib targeting the EGFR signaling pathway (**Figure 7D**), Palbociclib targeting CDK4/6 (**Figure 7C**), and Venetoclax targeting apoptosis regulation (**Figure 7E**). Analyzing the correlation between specific 33 bacterial species and the sensitivity to these targeted drugs revealed that several bacteria showed a positive correlation with the IC50 trends of targeted drugs such as KU.55933, BMS.345541 (12/33), MG.132 (8/33), NVP.ADW742 (16/33), and BIBR.1532 (9/33) (**Figure 7B**). This suggests that patients with a high PRIBR Index may have a poor response to targeted therapy.

## 4 Discussion

In this study, we aimed to establish a prognosis-related machine learning predictive model for intra-tumoral microbiota in low-grade gliomas, seeking to identify the multi-omic differences of intra-tumoral microbiota in different patterns of LGG. This study represents the first comprehensive analysis of the differential impacts caused by microbial infiltration in gliomas. we shed light on the effects of microbial infiltration on patient prognosis, grading, mRNA transcription, potential pathway variances, epigenetic alterations, genomic characteristics, and drug sensitivity in gliomas. This study addresses a significant gap in the current understanding of this field.

Over the past century, there has been a growing recognition of the role of microorganisms in the development of tumors. Initially, certain viruses capable of inducing cancer, such as the Epstein-Barr virus, human papillomavirus, and hepatitis B virus, were discovered. In the late twentieth century, the discovery of Helicobacter pylori in the stomach led to discussions in the academic community about the role of bacteria in tumors (42). With the emergence of 16s rRNA sequencing and metagenomic next-generation sequencing (mNGS) technologies, researchers can now independently sequence bacterial DNA. The first reports focused on the role of gut microbiota in the development of tumors, sparking significant interest among researchers in this field (43–45). The microbiota in the gut not only influences the biological functions of gastrointestinal tumors but also affects distant tumors by regulating the host’s systemic immune response (46). In 2020, Nejma et al. published a study in *Science* identifying and analyzing tumor microbiomes in seven different types of tumors. They identified associations between tumor-associated microorganisms and tumor subtypes in breast cancer, lung cancer, and glioblastoma, while immunotherapy responses were reported only in melanoma (23).

By analyzing the distribution of bacterial species and phyla within LGG tumors, we found significant differences in the bacterial infiltration status among different samples. The abundance of *Proteobacteria* was highest in most samples, with *Pseudomonas* being detectable in most samples, consistent with the sequencing results reported by Nejma et al. in high-grade gliomas (23). Subsequently, we employed 60 different machine learning algorithms to construct various models related to LGG-associated microbiota and validated them. We selected the algorithm with the highest accuracy to establish the model. Ultimately, we identified 33 bacteria associated with the survival period and status of LGG patients. The risk score based on the abundance of these 33 bacteria exhibited significant differences among different clinical groups and could serve as an independent prognostic factor.

In the high-risk group, there is infiltration by a greater variety of bacterial species. To identify the potential impact of tumor microbiota infiltration status under different patterns on the biological behavior of LGG, we conducted differential analysis of mRNA transcription profiles between high and low-risk groups identified by the model. The results were unexpected, as almost all differentially expressed genes exhibited a higher expression pattern in the high-risk group. Enrichment analysis of these genes revealed enrichment in chemokine or T-cell pathways using different analysis methods, implying that microbiota within tumors may be involved in the tumor’s immune status. Our subsequent analysis of the tumor microenvironment similarly validated this point, showing increased T-cell infiltration in the tumor microenvironment of the high-risk group. In drug sensitivity analysis, we found that LGG patients in the high-risk group benefited less from certain targeted drugs in sensitivity predictions.

Some viewpoints suggest that protective immune responses against certain pathogens may also target tumors (47), as evidenced by studies on the response of certain gut microbiota to immune checkpoint inhibitors in tumors (48, 49). Therefore, we attempted to analyze the expression of chemokines between the high and low-risk groups. The results showed that the differentially expressed chemokines were upregulated in the high-risk group, indicating that tumors and bacteria may jointly induce immune responses. However, in gliomas, the higher the identified bacterial infiltration associated with prognosis, the shorter the survival time of glioma patients. This phenomenon contradicts existing theories. Thus, we attempted to analyze certain immune checkpoints such as CD274 (PDL1), CD48, and HAVCR2 (TIM-3) (50–52), which have been proven to play negative roles in gliomas. The expression levels of these immune checkpoints were higher in the high-risk group, which may partially explain the contradictory findings compared to previous research results.

At the same time, we also attempted to analyze whether this different bacterial infiltration status would affect the genomic and epigenetic modification status of tumor cells. At the genomic level, there seemed to be little difference between the high and low-risk groups, with only the mutation rate of EGFR being more common in the high-risk group compared to the low-risk group. However, the analysis of methylation status showed that methylation status on multiple pathways appeared to change in the high-risk group, which may be related to toxins released by certain bacteria (53). The symbiotic relationship between cells and bacteria can also alter the epigenetic modification status of cells (41, 54). Takashi et al.’s study demonstrated that the gene methylation level of TLR4 in intestinal epithelial cells was higher in conventional mice compared to germ-free mice (41). This result partly explains the reason for the phenotypic differences between samples with different cell-bacteria symbiotic relationships in LGG.

We are the first to analyze the relationship between different microbial states within glioma samples and multi-omics differences in tumors. Despite obtaining many meaningful results, our study has certain limitations. Firstly, the microbial data we relied on is from the online BIC database, which only includes LGG samples, this cohort only includes samples classified as WHO grade 2 and 3. This means that although previous research tends to focus more on glioblastoma (28), we had to exclude these samples. Secondly, although we included 507 samples from online databases in our study, clinical sample validation may also require large-scale sequencing datasets to avoid biases. Due to time constraints, we did not include more clinical samples for validation. For the clinical samples included in this study, we performed 16S rRNA and proteomic sequencing. However, due to the limited sample size, additional analyses (e.g., single cell sequencing or histological staining) were not feasible. In addition, the bacterial infiltration abundance data we obtained were derived from unmapped reads in TCGA sequencing results, which were not assigned to specific genes. Compared to results obtained from 16S rRNA sequencing, this method inherently carries certain potential biases. However, given the lack of large-scale 16S rRNA sequencing data specifically targeting gliomas, we had no choice but to adopt this approach. Similar to previously published studies on intratumoral bacterial infiltration, our machine learning-based analyses were conducted using the relative abundance of bacterial infiltration. This may raise concerns among some researchers. To address these potential concerns, we supplemented our findings with bacterial sequence counts measured in clinically derived glioma samples. It is important to note that, whereas the BIC database leverages normal brain samples from TCGA as controls, our 16S rRNA sequencing analysis—conducted under ethical constraints—was restricted to tumor specimens and did not include matched normal brain tissue. This limitation may have introduced bias into our validation results. Moreover, we used both EPIC and ESTIMATE algorithms to predict immune−cell infiltration. Because these approaches are based exclusively on bioinformatic inference and our study included only a limited number of clinically collected glioma samples, we were unable to validate the TCGA−derived findings in an independent external cohort. This limitation may introduce some degree of bias into our results. Lastly, while our analysis suggests that intra-tumoral bacteria are associated with various cellular activities, we also hope for large-scale microbiome studies targeting all glioma subtypes.

## Data Availability

The datasets presented in this study can be found in online repositories. The names of the repository/repositories and accession number(s) can be found in the article/**Supplementary Material**.
