# Supplementary figures and images for: Deciphering the impact of intra-tumoral bacterial infiltration on multi-omics profiles in low-grade gliomas
Source: Front Oncol. 2025 Jun 18;15:1582068. doi: 10.3389/fonc.2025.1582068 (PMC12213438; doi:10.3389/fonc.2025.1582068)

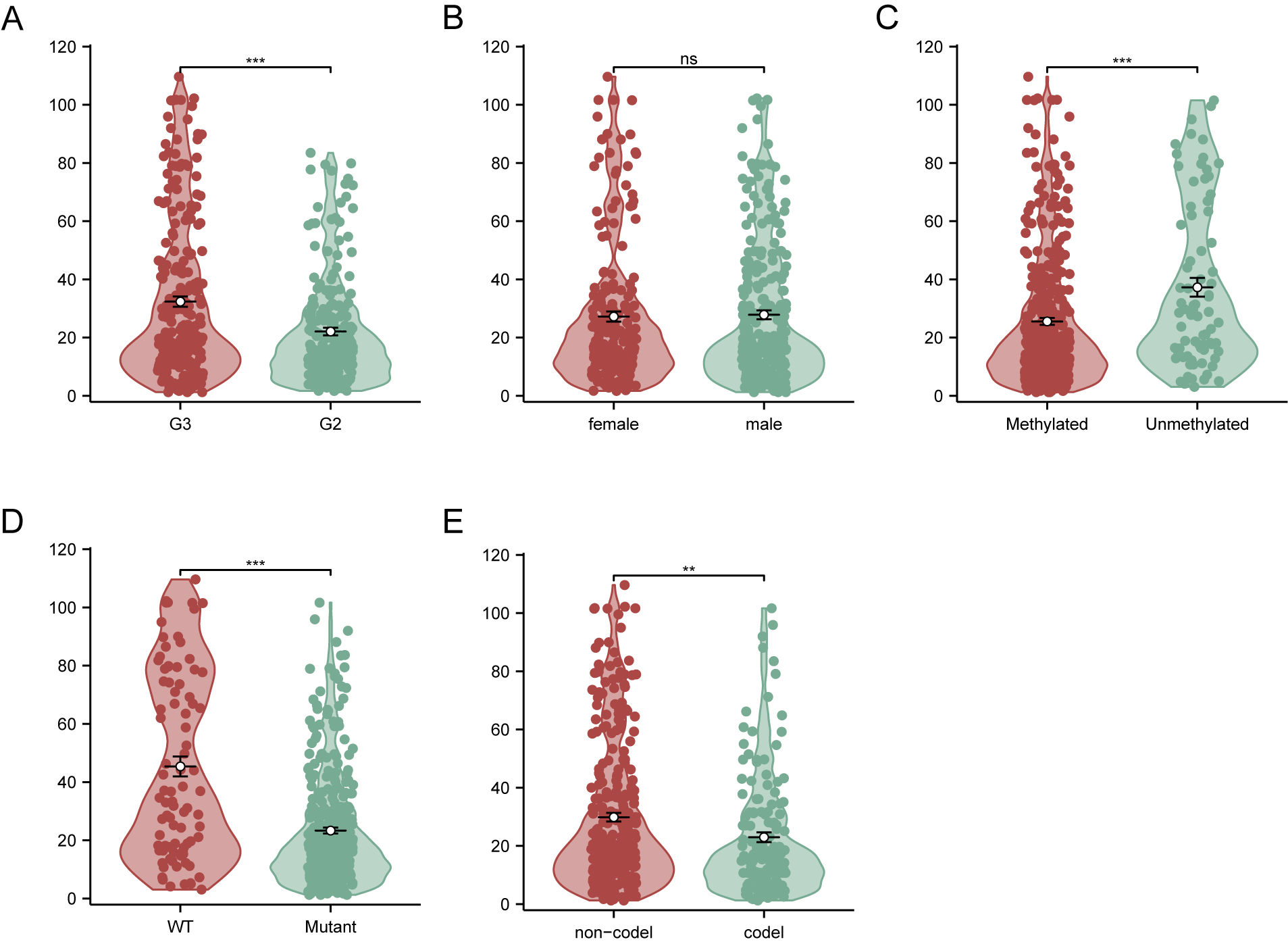

Supplement: Supplementary file 1 [file DataSheet1.zip › suppl.images and tables/FS1.tif]

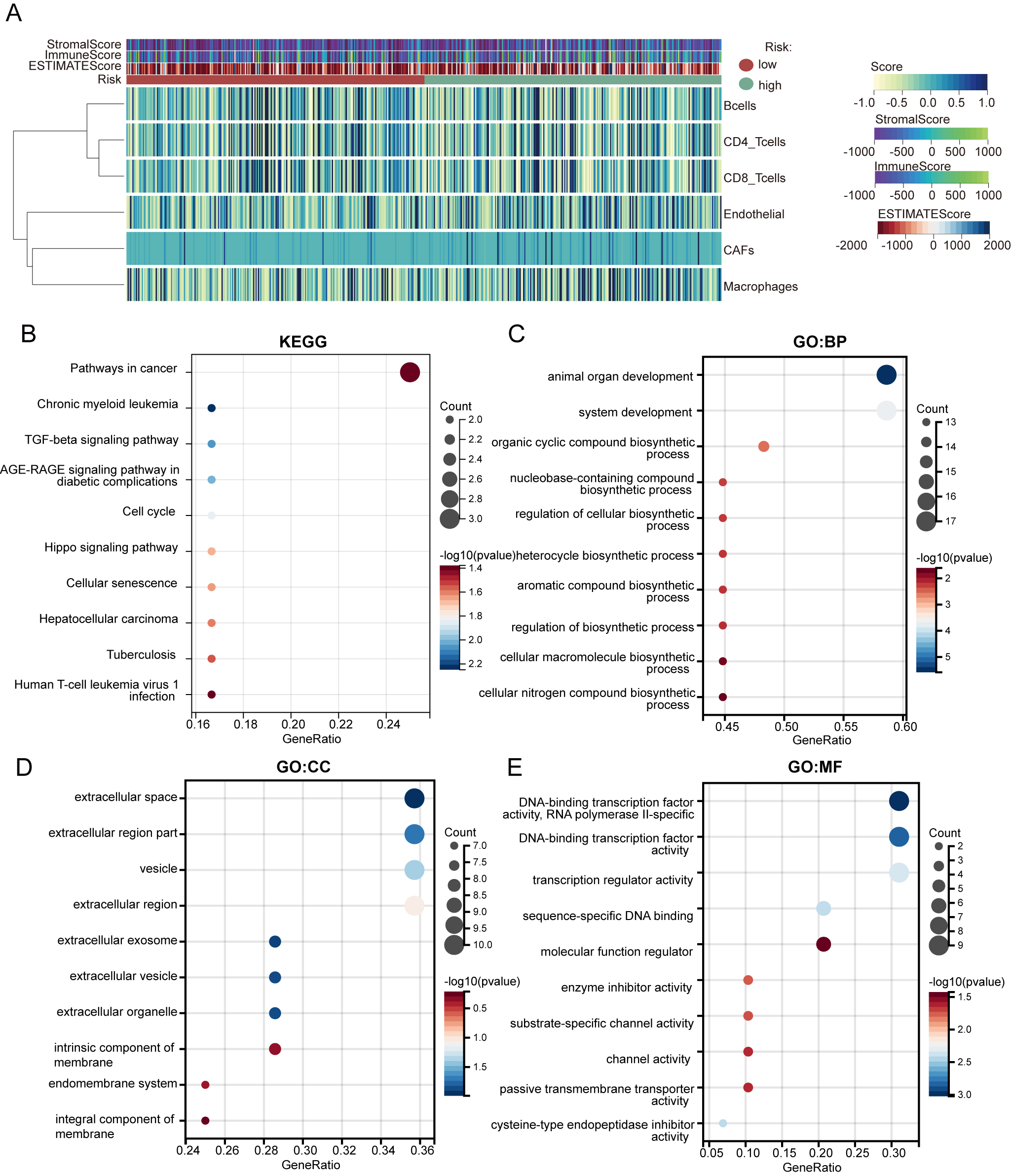

Supplement: Supplementary file 1 [file DataSheet1.zip › suppl.images and tables/FS2.tif]

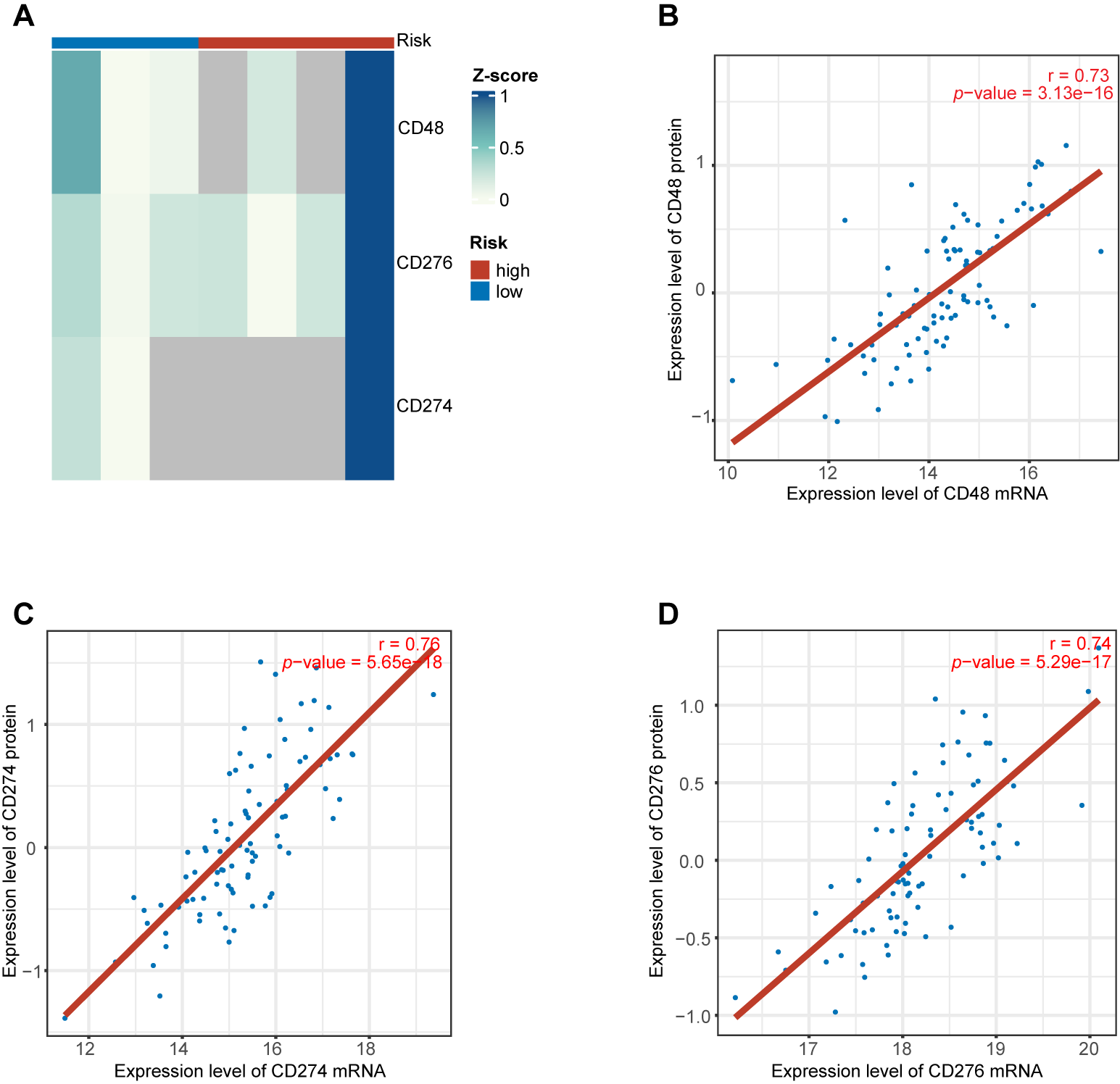

Supplement: Supplementary file 1 [file DataSheet1.zip › suppl.images and tables/FS3.tif]
